# Association of the dietary copper intake with all-cause and cardiovascular mortality: A prospective cohort study

**DOI:** 10.1371/journal.pone.0292759

**Published:** 2023-10-13

**Authors:** Lei Wang, Yun-Tao Zhao

**Affiliations:** Department of Cardiology, Aerospace Center Hospital, Haidian District, Beijing, PR China; Lithuanian University of Health Sciences, LITHUANIA

## Abstract

**Background:**

Copper (Cu) is a component that performs a crucial role in the normal function and development of the human body. Nonetheless, it is still largely unclear how Cu consumption in the diet relates to the risk for all-cause and cardiovascular disease (CVD) mortality.

**Methods:**

Data from the National Health and Nutrition Examination Survey from 2001–2018 were used to conduct a prospective cohort study of individuals between the ages of 20 years and above. Regression coefficients and 95% confidence intervals for the link between dietary Cu consumption and all-cause and cardiovascular-related mortality were computed utilizing univariate and multivariate-adjusted Cox proportional hazards models.

**Results:**

A total of 197.9 million non-institutionalized American citizens were represented by the NHANES’s 39,784 participants. The link between Cu in the diet and all-cause mortality was discovered to be non-linear in our restricted cubic spline regression models. When comparing the highest with the lowest quartile of Cu consumption in the diet, the weighted multivariate hazard ratios for all-cause mortality were 0.91 (0.83–0.99) for Q2, 0.88 (0.80–0.97) for Q3, and 0.86 (0.76–0.98) for Q4 (P for trend = 0.017). An identical trend was observed for cardiovascular mortality, but the association is not significant.

**Conclusion:**

The most important discovery was that higher dietary Cu consumption was associated with a lower risk of all-cause mortality. This trend was also consistent with that of cardiovascular-related mortality, but the association is not significant.

## Background

Minerals are very important in regulating the normal function of the body’s tissues and organ systems. Specifically, copper (Cu) is an important mineral micronutrient that performs a fundamental function in the metabolic processes that take place in cells. In addition to these roles, Cu serves as a regulatory agent, an integrated structural component, and a constituent of various cuproenzymes. However, it has been found that elevated Cu levels could enhance the synthesis of reactive oxygen species (ROS), leading to the oxidation of proteins, lipids, DNA, and other molecules [1, 2]. Because of its critical role, there is an increasing interest in the health effects of Cu intake [3].

Cu is an essential micronutrient human health, but like many nutrients, it must be consumed in appropriate amounts to avoid adverse health effects. Several previous studies have investigated the association between blood Cu concentration and the risk of all-cause death, but the results have been inconsistent [1, 4]. Previous studies that investigated the relationship between dietary Cu intake and mortality also reported mixed results [5, 6]. A recent study in general Chinese adults found that the association between dietary Cu intake and all-cause mortality followed a J-shape [7].

The correlations of trace minerals with the risk of developing CVD have gained a considerable deal of interest in recent years [1, 8–10]. There are controversies on the impact of the consumption of certain metals in one’s diet on risk factors and CVD-related events [11]. Numerous research reports have pointed to a correlation between levels of metals and risk factors and events associated with CVD [8, 12–14], while other studies show little indication that Cu has an independent influence on the biological markers that predict CVD risk [8, 10, 11]. Interestingly, one case-control study illustrated that higher consumption of Cu in the diet was linked to a reduced incidence of stroke [15].

To bridge this knowledge gap, there has to be more research done on a large scale over an extended time on the influence of trace mineral consumption on all-cause mortality and cardiovascular events in the general population. Therefore, this research sought to examine the link between Cu consumption and all-cause and cardiovascular-related mortality.

## Methods

### Ethics approval and consent to participate

This was a prospective cohort study using data from the US National Health and Nutrition Examination Survey (NHANES). The National Center for Health Statistics (NCHS) Research Ethics Review Board approved the underlying protocol and written informed consent was obtained from all participants. The de-identified data are freely available at the NHANES website (https://www.cdc.gov/nchs/nhanes.htm) and local ethics committee approval is not required for secondary analyses.

### Research design and participants

Data from the National Health and Nutrition Examination Survey (NHANES) from 2001–2018 were evaluated prospectively on individuals between the ages of 20 years and above. The US NHANES employed a multi-stage, rigorous probability sampling procedure to acquire health statistics that were representative of the American people.

The database includes demographics data, dietary data, examination data, laboratory data and questionnaire data. The interview section covers demographic, socioeconomic, diet and health-related issues; The physical examination section includes basic medical information, including blood pressure, audiometry, oral health, grip strength, etc., as well as a number of laboratory tests and some radiology data. Like previous health screening surveys, the study collected data on the prevalence of chronic diseases in the population. Such information is a particular advantage of the NHANES program. The investigation includes examining risk factors, that is, factors in a person’s lifestyle, constitution, genetics or environment that may increase the chance of developing a disease or condition, such as smoking, alcohol consumption, sexual behaviour, drug use, physical health and activity, weight and dietary intake. The details of the NHANES laboratory or medical technologist, as well as anthropometry procedures, were demonstrated in a previous study [16].

### Baseline data collection

Two 24-hour dietary-recall interviews are available to all NHANES participants. The NHANES database includes both detailed information on specific foods consumed and aggregate data on nutrient consumption. For this research, we used information from the Total nutrient Intake archive: the total amount of energy and nutrients consumed from food and drink per day for each participant. Cu intake (in mg) data are available on the NHANES portal. The average Cu intake of the two 24-h recalls was used for analysis.

Baseline data were obtained through questionnaires and comprised race/ethnicity, smoking status, age, marital status, sex, income poverty ratio for households, baseline medical history based on self-reporting (e.g., CVD, diabetes, hypercholesterolemia, hypertension), and medications (antihypertensive, hypoglycemic, lipid-lowering, etc.). Body mass index (BMI) is derived from a measure of weight and height. Standard protocols for taking measurements in the lab were used. The NHANES protocol (https://wwwn.cdc.gov/nchs/nhanes/analyticguidelines.aspx) details the steps used to investigate and compile data on clinical laboratory and procedure availability. Below are the criteria used to describe smoking status: never smoked—have a lifetime cigarette smoking count of fewer than one hundred; former smokers—have given up smoking after consuming > 100 cigarettes; current smokers—have > 100 cigarette smoking counts on various days, if not daily, during their lifetime. Meanwhile, alcohol consumption was defined based on the following criteria: current heavy drinker (≥3 drinks/day); current moderate alcohol user (≥2 drinks/day and <3 drinks/day); or current mild alcohol user (not conform to the aforementioned criteria).

For diabetes, the following groups were established: diabetes mellitus (DM); impaired glucose tolerance, or compromised fasting glycemia. In addition, the following indicators of diagnosis were applied: self-reported diagnosis of diabetes; medications for diabetic treatment; hemoglobin A1c level ≥6.5%; fasting plasma glucose ≥7.0 mmol/L (126 mg/dL); 2-h oral glucose tolerance test blood sugar ≥11.1 mmol/L (200 mg/dL), random plasma blood glucose ≥11.1 mmol/L (200 mg/dL) [17]. Meanwhile, clinical definition for hypertension evaluation included: a self-reported hypertension diagnosis; treatment with anti-hypertensive drugs; average systolic blood pressure ≥140 mmHg; and/or average diastolic blood pressure ≥90 mmHg [18, 19].

Participants’ CVD status was determined depending on their self-diagnoses of one or more of the following 5 CVD phenotypes: stroke, congestive heart failure, myocardial infarction, angina pectoris, and coronary artery disease. Participants were classified as having CVD if they reported having at least one health problem that was self-reported as "positive", and those with CVD could fit the criteria for more than one of the above subtypes.

Finally, chronic kidney disease was described as aberrant renal function as per kidney disease: Improving Global Outcomes 2021 clinical practice guidelines [20].

### Mortality

De-identified and anonymous participant data from NHANES were linked to Medicare and death records throughout the years 2001–2018 using the sequence numbers issued to each participant. Research participants’ mortality was tracked from the survey’s inception through December 31, 2019. All-cause mortality and mortality linked to cardiac diseases (I00-I09, I11, I13, and I20-I51), DM (E10-E14), chronic lower respiratory illness (J40-J47), malignancies (C00-C97), cerebrovascular disease (I60-I69), Alzheimer’s disease (G30), and other causes were analyzed. The 10th version of the International Classification of Diseases (ICD-10) was used to establish criteria for cardiovascular-related mortality. It includes deaths due to heart diseases (I00-I09, I11, I13, and I20-I51) and cerebrovascular diseases (I60–169), and it also helps in identifying the cause of death events.

### Statistical analysis

Both the mean ± SD for normally distributed and the median and interquartile range for skewed data were used for presenting continuous variables, while numbers or percentages were employed to present categorical variables. Two-sample t-tests (categorical variables), one-way analysis of variance (ANOVA) (normal distribution), and the Kruskal-Wallis H-test (skewed distribution) were implemented to examine differences (variations) among dietary Cu consumption quartiles. Each variable was checked for any missing pieces before the data was analyzed. CKD accounted for the vast majority of the incomplete details (0.00–26.0%), hence, dummy variables were used to represent absent covariates values during analyses. We employed the restricted cubic spline model to evaluate the shape of the association between Cu in the diet and death from any cause and CVDs. The knots in the 25^th^, 50^th^, and 75^th^ quartiles were chosen. In addition, Cox proportional hazard models were estimated for Cu consumption after controlling for confounding variables, and regression coefficients and 95% CIs were used to describe the results. Regression models were estimated for the total sample and corrected for confounders such as demographic, socioeconomic, behavioral, physical examination, and medical history. A P < 0.05 indicated a significance level.

### Sensitivity analysis

Subgroup analyses were carried out in terms of gender (male or female), age at the beginning of the cohort (less than or more than 65 years old), and health history (hypertension and diabetes). Dietary Cu intake was estimated by a multivariate-adjusted Cox proportional hazards model, and regression coefficients and 95% Cis were employed to describe the findings. In addition, R 4.1.2 (http://www.r-project.org) was adopted for all analyses of statistical data and modified for complicated survey design and population weighting according to the survey protocol.

## Results

The 39,784 people who took part in the NHANES constituted a representative sample of 197,9 million non-institutionalized American people. The participants belonged to the age group of 47.6 ± 17.0 years and were primarily composed of 52.6% women, 69.3% non-Hispanic white, 11.2% non-Hispanic black, and 7.9% Mexican Americans (Table 1).

**Table 1:** The weighted features of the subjects were stratified based on their dietary Cu intake quartiles.

	Level	Overall	Cu Intake Quartile, mg/d				*p*
			Q1 (<0.8)	Q2 (≥0.8 to <1.1)	Q3 (≥1.1 to <1.5)	Q4 (≥1.5)	
N		197934248	39569101	50679649	53829823	53855676	
Age (mean (SD))		47.6 (17.0)	48.2 (18.3)	48.5 (17.6)	47.7 (16.7)	46.3 (15.7)	<0.001
Sex (%)	Female	104133861 (52.6)	27470788 (69.4)	30353025 (59.9)	26878787 (49.9)	19431261 (36.1)	<0.001
	Male	93800387 (47.4)	12098313 (30.6)	20326624 (40.1)	26951035 (50.1)	34424415 (63.9)	
BMI (mean (SD))		28.91 (6.78)	29.40 (7.28)	29.11 (6.78)	28.99 (6.69)	28.27 (6.43)	<0.001
Race/ethnicity (%)	Mexican American	15640783 (7.9)	2922696 (7.4)	4251243 (8.4)	4359783 (8.1)	4107061 (7.6)	<0.001
	Non-Hispanic Black	22148363 (11.2)	7134320 (18.0)	5958632 (11.8)	5112520 (9.5)	3942892 (7.3)	
	Non-Hispanic White	137150388 (69.3)	24865916 (62.8)	34662846 (68.4)	38292229 (71.1)	39329397 (73.0)	
	Other Race	22994714 (11.6)	4646169 (11.7)	5806928 (11.5)	6065291 (11.3)	6476326 (12.0)	
Education (%)	College or above	119522596 (60.4)	18457381 (46.6)	28803044 (56.8)	34297155 (63.7)	37965017 (70.5)	<0.001
	High school or equivalent	68060404 (34.4)	17951613 (45.4)	18954660 (37.4)	16966103 (31.5)	14188029 (26.3)	
	Less than high school	10235094 (5.2)	3132880 (7.9)	2877520 (5.7)	2538556 (4.7)	1686137 (3.1)	
Marital status (%)	Married	110594350 (55.9)	18214330 (46.0)	27882150 (55.0)	31876879 (59.2)	32620992 (60.6)	<0.001
	Never married	50064491 (25.3)	11503119 (29.1)	12504369 (24.7)	12814978 (23.8)	13242026 (24.6)	
	Separated	37187523 (18.8)	9846323 (24.9)	10273080 (20.3)	9092818 (16.9)	7975302 (14.8)	
Family income-poverty ratio (mean (SD))		3.02 (1.64)	2.46 (1.60)	2.93 (1.61)	3.17 (1.61)	3.36 (1.60)	<0.001
Smoking status (%)	Never	107053139 (54.1)	19608640 (49.6)	27491791 (54.2)	30014072 (55.8)	29938637 (55.6)	<0.001
	Former	50285037 (25.4)	8431898 (21.3)	12633046 (24.9)	14220538 (26.4)	14999555 (27.9)	
	Now	40524253 (20.5)	11509216 (29.1)	10538341 (20.8)	9568503 (17.8)	8908193 (16.5)	
Alcohol use (%)	Never	21394155 (10.8)	5563308 (14.1)	5623485 (11.1)	5374606 (10.0)	4832756 (9.0)	<0.001
	Mild	67476963 (34.1)	10202233 (25.8)	16838412 (33.2)	19096086 (35.5)	21340232 (39.6)	
	Moderate	30536565 (15.4)	6400665 (16.2)	7761535 (15.3)	8622588 (16.0)	7751777 (14.4)	
	Heavy	38546252 (19.5)	7930885 (20.0)	9975770 (19.7)	10252423 (19.0)	10387174 (19.3)	
	Former	27225808 (13.8)	6310141 (15.9)	7046799 (13.9)	7362012 (13.7)	6506857 (12.1)	
DM (%)	No	156127504 (78.9)	30867280 (78.0)	39046868 (77.0)	42326858 (78.6)	43886498 (81.5)	<0.001
	DM	26005509 (13.1)	5904840 (14.9)	7478320 (14.8)	7007076 (13.0)	5615273 (10.4)	
	IFG	7807362 (3.9)	1536265 (3.9)	1899769 (3.7)	2224199 (4.1)	2147130 (4.0)	
	IGT	5192918 (2.6)	820057 (2.1)	1565066 (3.1)	1414326 (2.6)	1393468 (2.6)	
Hypertension (%)	No	122404990 (61.8)	22874225 (57.8)	30601130 (60.4)	33321432 (61.9)	35608202 (66.1)	<0.001
	Yes	75529258 (38.2)	16694876 (42.2)	20078519 (39.6)	20508391 (38.1)	18247473 (33.9)	
CVD (%)	No	179942897 (90.9)	34835208 (88.0)	45667457 (90.1)	49390311 (91.8)	50049921 (92.9)	<0.001
	Yes	17975161 (9.1)	4731274 (12.0)	5012192 (9.9)	4438914 (8.2)	3792781 (7.0)	
Hyperlipidemia (%)	No	60443286 (30.5)	11380381 (28.8)	14700748 (29.0)	16368171 (30.4)	17993987 (33.4)	<0.001
	Yes	137465064 (69.4)	28168618 (71.2)	35977516 (71.0)	37457240 (69.6)	35861689 (66.6)	
CKD (%)	No	125236363 (63.3)	22658496 (57.3)	31406875 (62.0)	35662291 (66.3)	35508701 (65.9)	<0.001
	Yes	22357928 (11.3)	5661449 (14.3)	6515863 (12.9)	5609740 (10.4)	4570876 (8.5)	

The results are displayed as either frequency (in percentages), means (with standard deviations), or medians (IQR). The absence of some data is the cause of the numbers not adding up to a total of 100%. Abbreviations: BMI, body mass index (weight in kg/height in m^2^); CKD, chronic kidney disease; CVD, cardiovascular disease; DM, diabetes mellitus; IGT, impaired glucose tolerance; IFG, impaired fasting glycemia.

Table 1 provides an overview of the weighted baseline characteristics of the people who took part in the research. These characteristics are stratified according to the quartiles of the individuals’ dietary Cu consumption. There was a significant disparity in the ages between the quartiles of dietary Cu consumption (P < 0.001), with the individuals in the 4th quartile having an average age of 46.3 ± 15.7 years, which was lower than the ages of those in the other quartiles. Additionally, the male inclination was shown in the 4th quartile, whilst the female inclination was seen in the 1st quartile of the distribution (P < 0.001). In addition, the percentage of non-Hispanic whites was rather elevated in the 3rd and 4th quartiles (P < 0.001). In the 4th quartile, there was a greater proportion of married individuals (60.6%, P < 0.001), as well as people whose household income-to-poverty ratios were relatively high (P < 0.001). The majority of individuals who met the criteria for "college or above" education level were in the 4th quartile (70.5%, P < 0.001), and the 12% of patients in the 1st quartile had a substantially greater percentage of pre-existing cardiovascular disease at the time of the study (P < 0.001).

**Fig 1:**
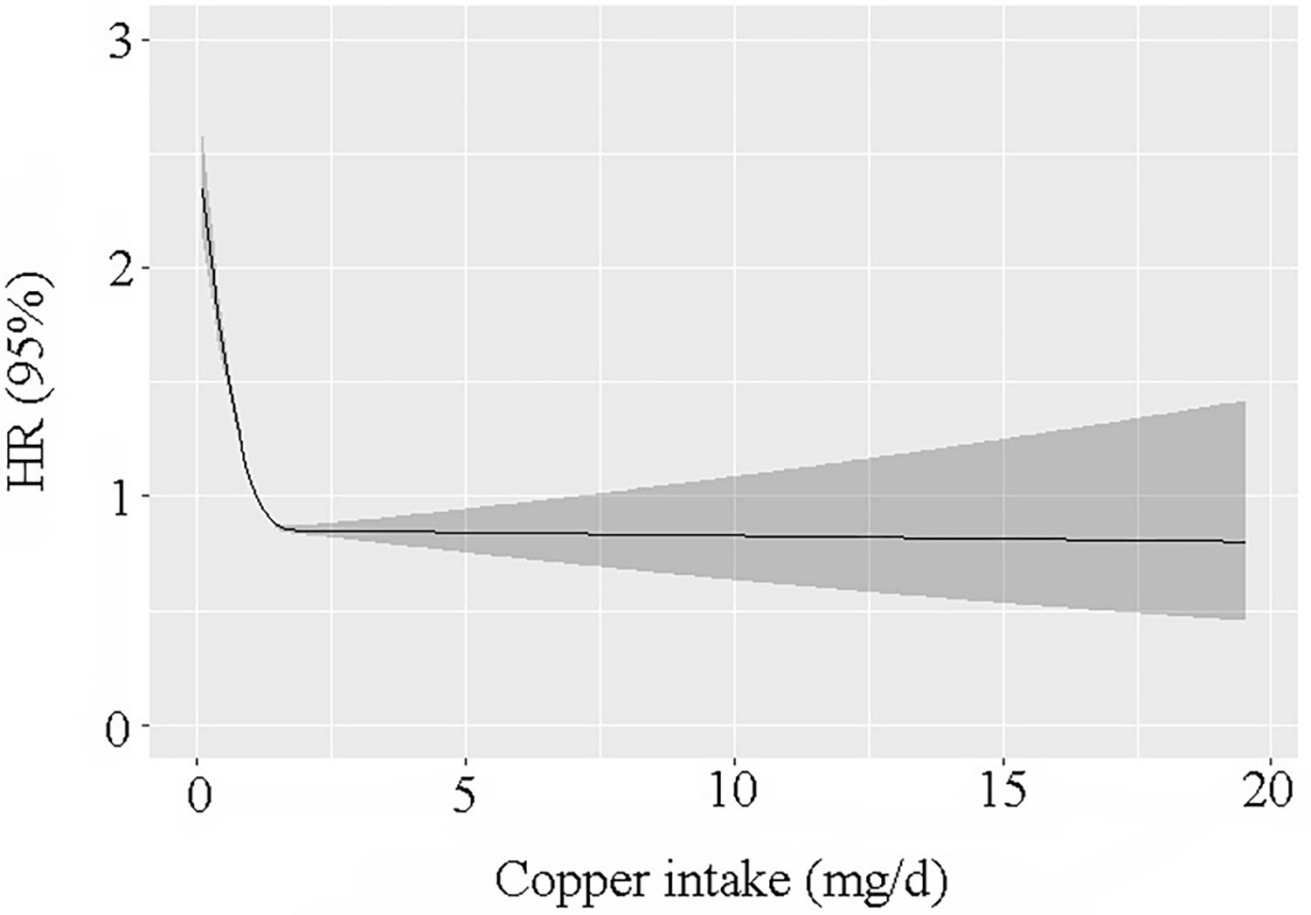
Generalized additive models were applied to show the link between dietary Cu consumption in the diet and all-cause mortality.

**Fig 2:**
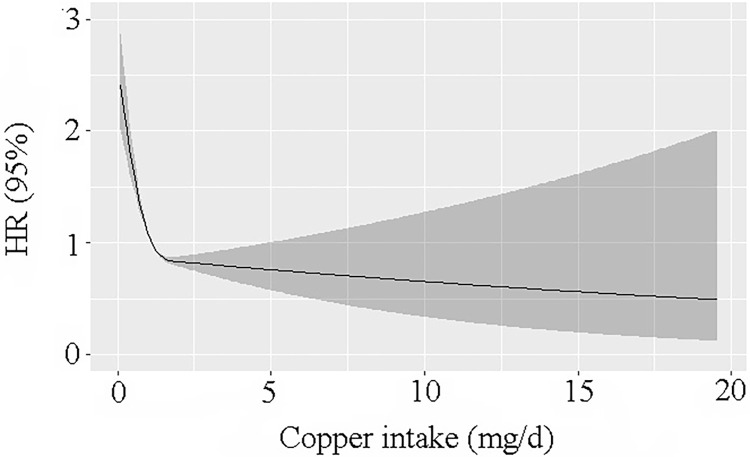
Generalized additive models were employed to show the link between Cu consumption in the diet and cardiovascular-related mortality.

During the follow-up time duration of 110 months, 5,753 death events were recorded, with 1,447 of those deaths attributable to CVDs and 312 of those deaths attributable to cerebrovascular illness. As per the outcomes of the restricted cubic spline regression models, we discovered that the link between the amount of Cu in one’s diet and death from any causes was not linear (Fig 1). In addition, there was an L-shaped link between the amount of Cu in a person’s diet and all-cause mortality. There was a non-linear association between the amount of Cu in the diet and the risk of all-cause mortality, and the risk dropped as the amount of Cu increased (adjusted R2 = 0.008, Dxy = 0.146, P for non-linearity < 0.001). Similarly, a non-linear association was recorded between an increase in dietary Cu consumption and a reduction in the risk of death from cardiovascular-related causes (adjusted R2 = 0.004, Dxy = 0.168, P for non-linearity < 0.001; Fig 2).

The connection between dietary Cu consumption and death from any causes was 0.78 in the unadjusted, weighted model (0.71, 0.86) (S1 Table). Elevated Cu intake was shown to be related to a decreased risk of all-cause mortality after accounting for lab test results, drugs, medical history, behavior, anthropometric variables, socioeconomics, and demographics (Model 5). We also found that the weighted multivariate HR values for all-cause mortality were 0.91 (0.83–0.99) for Q2, 0.88 (0.80–0.97) for Q3, and 0.86 (0.76–0.98) for Q4 (P for trend = 0.017; Table 2).

**Table 2:** Weighted associations between the dietary Cu intake and all-cause and cardiovascular mortality in the multivariable, and crude analyses.

Cu	Q1 (<0.8)	Q2 (≥0.8 to <1.1)	Q3 (≥1.1 to <1.5)	Q4 (≥1.5)	*P* Value for Trend
All- cause mortality					
Model1	1	0.80 (0.73,0.88) <0.001	0.66 (0.60,0.72) <0.001	0.51 (0.46,0.58) <0.001	<0.001
Model2	1	0.77 (0.70–0.85) 0.008	0.61 (0.56–0.67) <0.001	0.45(0.40–0.51) <0.001	<0.001
Model3	1	0.78 (0.72–0.85) 0.001	0.69 (0.64–0.75) <0.001	0.61 (0.55–0.69) <0.001	<0.001
Model4	1	0.87 (0.80–0.95) 0.001	0.81 (0.74–0.89) <0.001	0.75 (0.66–0.85) <0.001	<0.001
Model5	1	0.91 (0.83–0.99) 0.031	0.88 (0.80–0.97) 0.008	0.86 (0.76–0.98) 0.028	0.017
Cardiovascular mortality					
Model1	1	0.80 (0.69,0.94) 0.006	0.70 (0.59,0.83) <0.001	0.44 (0.35,0.56) <0.001	<0.001
Model2	1	0.77 (0.66–0.90) 0.001	0.64 (0.54–0.76) <0.001	0.38(0.30–0.48) <0.001	<0.001
Model3	1	0.79 (0.68–0.92) 0.003	0.74 (0.63–0.88) <0.001	0.56 (0.45–0.70) <0.001	<0.001
Model4	1	0.89 (0.76–1.04) 0.136	0.88 (0.73–1.04) 0.140	0.68 (0.54–0.86) 0.001	0.001
Model5	1	0.92 (0.78–1.08) 0.312	0.95 (0.80–1.13) 0.584	0.80 (0.63–1.02) 0.070	0.095

Data are hazard ratio (95% CI)

Model 1 unadjusted.

Model 2 adjusted for sex

Model 3 adjusted for age, sex.

Model 4 adjusted for model 3 covariates plus ethnicity, family income-poverty ratio level, education, and marital status.

Model 5 adjusted for model 4 covariates plus smoking status, diabetes, hypertension, CVD, and CKD.

Abbreviations:

HR, hazard ratio

CI, confidence interval

CVD, cardiovascular disease

CKD, chronic kidney disease

Meanwhile, the coefficient of determination for the relationship between dietary CU consumption and death due to cardiovascular-associated causes was 0.70 within the context of the weighted unadjusted model (0.60, 0.83) (S2 Table). Elevated Cu intake was shown to be related to a lower risk of death from cardiovascular-related causes after accounting for lab test results, drugs, medical history, behavior, anthropometric variables, socioeconomics, and demographics (Model 5). A comparative analysis of the lowest dietary Cu intake quartile illustrated that the weighted multivariate HRs for death from cardiovascular-related causes were 0.92 (0.78–1.08) for Q2, 0.95 (0.80–1.13) for Q3, and 0.80 (0.63–1.02) for Q4, and the association is not significant (P for trend = 0.095; Table 2).

In addition, we conducted subgroup analyses to investigate the association between dietary Cu consumption and all-cause mortality and CVD, stratifying the results according to age, gender, and previous medical conditions (S3 Table). There was no remarkable interplay between the subgroup characteristics and the association between dietary Cu consumption and all-cause mortality and CVDs.

## Discussion

By combining and processing NHANES data spanning the years 2001 through 2018, this extensive prospective research investigated the possible link between dietary Cu consumption and the risk of all-cause and cardiovascular-related mortality. In this report, participants whose dietary Cu intake was in the highest quartile recorded a lower risk of death from any cause compared to those whose intake was in the lowest quartile. This trend was also consistent with that of cardiovascular-related mortality. But the association is not significant between dietary Cu consumption and mortality from CVD risk.

Some previous studies have reported the effect of dietary Cu intake on the risk of all-cause death, but have reported inconsistent results [5, 6]. The previous study conducted in the Warsaw area between spring 1999 and December 31, 2003 found a higher all-cause mortality among 146 male participants in a subgroup of older men with lower Cu intake collected using three-dimensional recording methods [21]. Few studies have used dietary Cu intake data continuously, which may allow to present a non-linear relationship between Cu intake and all-cause mortality and provide more fine-grained information. Using a prospective cohort design, a relatively long follow-up period, and two consecutive 24-hour dietary reviews, our current study examined the association between adjusted dietary Cu intake and all-cause mortality in the U.S. general population by relatively comprehensive adjustment for a number of potential confounders.

Cu is a crucial transition metal required for a wide variety of vital eukaryotic functions, including lipid metabolism, redox equilibrium, and others [22]. Data from dietary surveys indicate a possible link between metabolic disorders and inadequate dietary Cu intake [23–25]. Previous studies have found that total mortality, primary vascular mortality, cancer mortality, and respiratory mortality in elderly people in the UK are predicted by several biochemical measures of oxidation-regulating nutrients [6]. Micronutrients perform fundamental functions in overall health maintenance, but like many nutrients, it must be consumed in appropriate amounts to avoid adverse health effects.

We acknowledge certain limitations in our present study. First, due to the observational nature of the research, we were unable to collect information on whether or not participants changed their diet or their behavior (including their exercise or sleeping behaviors). The diagnosis of CVD based on the self-reports from participants, this may lead to discrepancies from the actual situation. Moreover, inaccuracies in reflecting long-term food choices might exist since the dietary Cu intake was calculated only at baseline. Hence, to further validate our findings and reveal more insights into our results, further research in the form of prospective studies is needed.

## Conclusion

The most noteworthy result of this research was the discovery that elevated levels of Cu in the diet were linked to reduced risk of death from all causes combined. This trend was also consistent with that of cardiovascular-related mortality, but the association is not significant. Additional research is required to confirm these findings and shed light on their implications.

## Supporting information

S1 Table Weighted univariate cox regression model for all-cause mortality. (DOC)

S2 Table Weighted univariate cox regression model for cardiovascular mortality. (DOC)

S3 Table Weighted multivariable-adjusted hazard ratios for the association between quartiles of copper and all-cause mortality and cardiovascular mortality. (DOC)

## References

[1] Mohammadifard N, Humphries KH, Gotay C, Mena-Sanchez G, Salas-Salvado J, Esmaillzadeh A, et al. Trace minerals intake: Risks and benefits for cardiovascular health. Crit Rev Food Sci Nutr. 2019;59(8):1334–46. Epub 2017/12/14. doi: 10.1080/10408398.2017.1406332 . 29236516

[2] Fukai T, Ushio-Fukai M, Kaplan JH. Copper transporters and copper chaperones: roles in cardiovascular physiology and disease. Am J Physiol Cell Physiol. 2018;315(2):C186–C201. Epub 2018/06/07. doi: 10.1152/ajpcell.00132.2018 ; PubMed Central PMCID: PMC6139499. 29874110 PMC6139499

[3] Klevay LM. Copper Nutriture, a Hidden Variable in Cardiovascular Epidemiology. Curr Atheroscler Rep. 2019;21(7):24. Epub 2019/05/02. doi: 10.1007/s11883-019-0785-7 . 31041555

[4] Zabłocka-Słowińska K, Prescha A, Płaczkowska S, Porębska I, Kosacka M, Pawełczyk K. Serum and Whole Blood Cu and Zn Status in Predicting Mortality in Lung Cancer Patients. Nutrients. 2020;13(1). doi: 10.3390/nu13010060 33375477 PMC7824662

[5] Chen F, Du M, Blumberg JB, Ho Chui KK, Ruan M, Rogers G, et al. Association Among Dietary Supplement Use, Nutrient Intake, and Mortality Among U.S. Adults: A Cohort Study. Ann Intern Med. 2019;170(9):604–13. Epub 2019/04/09. doi: 10.7326/M18-2478 ; PubMed Central PMCID: PMC6736694. 30959527 PMC6736694

[6] Bates CJ, Hamer M, Mishra GD. Redox-modulatory vitamins and minerals that prospectively predict mortality in older British people: the National Diet and Nutrition Survey of people aged 65 years and over. British Journal of Nutrition. 2010;105(1):123–32. doi: 10.1017/s0007114510003053 20807458 PMC3361131

[7] Gan X, He P, Zhou C, Zu C, Meng Q, Liu M, et al. J-shaped association between dietary copper intake and all-cause mortality: a prospective cohort study in Chinese adults. Br J Nutr. 2023;129(11):1841–7. Epub 2022/09/02. doi: 10.1017/S0007114522002732 . 36047085

[8] Xu L, Liu Y, Zhao Q, Du H, Gao Y, Bai M, et al. Urinary element profiles and associations with cardiometabolic diseases: A cross-sectional study across ten areas in China. Environ Res. 2022;205:112535. Epub 2021/12/14. doi: 10.1016/j.envres.2021.112535 . 34896320

[9] Long P, Wang Q, Zhang Y, Zhu X, Yu K, Jiang H, et al. Profile of copper-associated DNA methylation and its association with incident acute coronary syndrome. Clin Epigenetics. 2021;13(1):19. Epub 2021/01/28. doi: 10.1186/s13148-021-01004-w ; PubMed Central PMCID: PMC7839231. 33499918 PMC7839231

[10] Palaniswamy S, Piltonen T, Koiranen M, Mazej D, Jarvelin MR, Abass K, et al. The association between blood copper concentration and biomarkers related to cardiovascular disease risk—analysis of 206 individuals in the Northern Finland Birth Cohort 1966. J Trace Elem Med Biol. 2019;51:12–8. Epub 2018/11/24. doi: 10.1016/j.jtemb.2018.09.003 . 30466920

[11] Malekahmadi M, Firouzi S, Rezayi M, Ghazizadeh H, Ranjbar G, Ferns GA, et al. Association of Zinc and Copper Status with Cardiovascular Diseases and their Assessment Methods: A Review Study. Mini Rev Med Chem. 2020;20(19):2067–78. Epub 2020/07/31. doi: 10.2174/1389557520666200729160416 . 32727323

[12] Eshak ES, Iso H, Yamagishi K, Maruyama K, Umesawa M, Tamakoshi A. Associations between copper and zinc intakes from diet and mortality from cardiovascular disease in a large population-based prospective cohort study. J Nutr Biochem. 2018;56:126–32. Epub 2018/03/13. doi: 10.1016/j.jnutbio.2018.02.008 . 29529560

[13] Liu YH, Wang CW, Wu DW, Lee WH, Chen YC, Li CH, et al. Association of Heavy Metals with Overall Mortality in a Taiwanese Population. Nutrients. 2021;13(6). Epub 2021/07/03. doi: 10.3390/nu13062070 ; PubMed Central PMCID: PMC8235372. 34204322 PMC8235372

[14] Grammer TB, Kleber ME, Silbernagel G, Pilz S, Scharnagl H, Lerchbaum E, et al. Copper, ceruloplasmin, and long-term cardiovascular and total mortality (the Ludwigshafen Risk and Cardiovascular Health Study). Free Radic Res. 2014;48(6):706–15. Epub 2014/03/13. doi: 10.3109/10715762.2014.901510 . 24605902

[15] Yang L, Chen X, Cheng H, Zhang L. Dietary Copper Intake and Risk of Stroke in Adults: A Case-Control Study Based on National Health and Nutrition Examination Survey 2013–2018. Nutrients. 2022;14(3). Epub 2022/03/13. doi: 10.3390/nu14030409 ; PubMed Central PMCID: PMC8839334. 35276768 PMC8839334

[16] Abramowitz MK, Hall CB, Amodu A, Sharma D, Androga L, Hawkins M. Muscle mass, BMI, and mortality among adults in the United States: A population-based cohort study. PLoS One. 2018;13(4):e0194697. Epub 2018/04/12. doi: 10.1371/journal.pone.0194697 ; PubMed Central PMCID: PMC5894968. 29641540 PMC5894968

[17] American Diabetes Association Professional Practice C. Classification and Diagnosis of Diabetes: Standards of Medical Care in Diabetes-2022. Diabetes Care. 2022;45(Suppl 1):S17–S38. Epub 2021/12/30. doi: 10.2337/dc22-S002 . 34964875

[18] Reboussin DM, Allen NB, Griswold ME, Guallar E, Hong Y, Lackland DT, et al. Systematic Review for the 2017 ACC/AHA/AAPA/ABC/ACPM/AGS/APhA/ASH/ASPC/NMA/PCNA Guideline for the Prevention, Detection, Evaluation, and Management of High Blood Pressure in Adults: A Report of the American College of Cardiology/American Heart Association Task Force on Clinical Practice Guidelines. J Am Coll Cardiol. 2018;71(19):2176–98. Epub 2017/11/18. doi: 10.1016/j.jacc.2017.11.004 ; PubMed Central PMCID: PMC8654280. 29146534 PMC8654280

[19] Flack JM, Adekola B. Blood pressure and the new ACC/AHA hypertension guidelines. Trends in Cardiovascular Medicine. 2020;30(3):160–4. doi: 10.1016/j.tcm.2019.05.003 31521481

[20] Kidney Disease: Improving Global Outcomes Glomerular Diseases Work G. KDIGO 2021 Clinical Practice Guideline for the Management of Glomerular Diseases. Kidney Int. 2021;100(4S):S1–S276. Epub 2021/09/25. doi: 10.1016/j.kint.2021.05.021 . 34556256

[21] Kałuza J, Dołowa J, Roszkowski W, Brzozowska A. Survival and habitual nutrient intake among elderly men. Roczniki Panstwowego Zakladu Higieny. 2005;56(4):361–70. Epub 2006/04/14. . 16610673

[22] Balsano C, Porcu C, Sideri S. Is copper a new target to counteract the progression of chronic diseases? Metallomics. 2018;10(12):1712–22. Epub 2018/10/20. doi: 10.1039/c8mt00219c . 30339169

[23] Morrell A, Tallino S, Yu L, Burkhead JL. The role of insufficient copper in lipid synthesis and fatty-liver disease. IUBMB Life. 2017;69(4):263–70. Epub 2017/03/09. doi: 10.1002/iub.1613 ; PubMed Central PMCID: PMC5619695. 28271632 PMC5619695

[24] Chen J, Jiang Y, Shi H, Peng Y, Fan X, Li C. The molecular mechanisms of copper metabolism and its roles in human diseases. Pflugers Arch. 2020;472(10):1415–29. Epub 2020/06/09. doi: 10.1007/s00424-020-02412-2 . 32506322

[25] Blades B, Ayton S, Hung YH, Bush AI, La Fontaine S. Copper and lipid metabolism: A reciprocal relationship. Biochim Biophys Acta Gen Subj. 2021;1865(11):129979. Epub 2021/08/09. doi: 10.1016/j.bbagen.2021.129979 . 34364973

