# Supplementary material for: Association of the dietary copper intake with all-cause and cardiovascular mortality: A prospective cohort study
Source: PLoS One. 2023 Oct 13;18(10):e0292759. doi: 10.1371/journal.pone.0292759 (PMC10575518; doi:10.1371/journal.pone.0292759)
Supplement: S1 Table — (DOC) [file pone.0292759.s001.doc]

|  | Level | HR (95%CI) | P |
| --- | --- | --- | --- |
| Age |  | 1.09 (1.09,1.10) | <0.001 |
| Sex | Female | 1 |  |
|  | Male | 1.22 (1.14,1.30) | <0.001 |
| Race/ethnicity | Mexican American | 1 |  |
|  | Non-Hispanic Black | 2.21 (1.84,2.65) | <0.001 |
|  | Non-Hispanic White | 2.37 (1.99,2.81) | <0.001 |
|  | Other Race | 1.26 (0.98,1.62) | 0.066 |
| Education | College or above | 1 |  |
|  | High school or equivalent | 1.77 (1.65,1.91) | <0.001 |
|  | Less than high school | 3.29 (2.90,3.72) | <0.001 |
| Marital status | Married | 1 |  |
|  | Never married | 0.58 (0.50,0.68) | <0.001 |
|  | Separated | 2.62 (2.42,2.83) | <0.001 |
| Family income-poverty ratio | <1.0 | 1 |  |
|  | 1.0-3.0 | 1.15 (1.04,1.28) | 0.009 |
|  | >3.0 | 0.55 (0.49,0.62) | <0.001 |
| BMI |  | 1.01 (1.00,1.01) | 0.053 |
| copper |  | 0.78 (0.71,0.86) | <0.001 |
|  | Q1 (<0.8) | 1 |  |
|  | Q2 (≥0.8 to <1.1) | 0.80 (0.73,0.88) | <0.001 |
|  | Q3 (≥1.1 to <1.5) | 0.66 (0.60,0.72) | <0.001 |
|  | Q4 (≥1.5) | 0.51 (0.46,0.58) | <0.001 |
| Smoking status | Never | 1 |  |
|  | Former | 2.26 (2.07,2.47) | <0.001 |
|  | Now | 1.48 (1.35,1.62) | <0.001 |
| Alcohol use | Never | 1 |  |
|  | Mild | 0.74 (0.64,0.87) | <0.001 |
|  | Moderate | 0.43 (0.36,0.52) | <0.001 |
|  | Heavy | 0.42 (0.34,0.51) | <0.001 |
|  | Former | 1.65 (1.42,1.92) | <0.001 |
| DM | No | 1 |  |
|  | DM | 3.76 (3.45,4.12) | <0.001 |
|  | IFG | 2.18 (1.89,2.51) | <0.001 |
|  | IGT | 1.90 (1.60,2.27) | <0.001 |
| Hypertension |  | 4.05 (3.72,4.41) | <0.001 |
| Hyperlipidemia |  | 1.59 (1.46,1.74) | <0.001 |
| CVD |  | 6.30 (5.78, 6.87) | <0.001 |
| CKD |  | 6.25 (5.75,6.80) | <0.001 |

S1 Table . Weighted univariate cox regression model for all-cause mortality

Abbreviations:

HR, hazard ratio

CI, confidence interval

BMI, the body-mass index is determined as follows: the weight in kilograms (Kgs) / (height in square meters (m^2^)

DM, diabetes mellitus

IFG, impaired fasting glycaemia

IGT impaired glucose tolerance

CVD, cardiovascular disease

CKD, chronic kidney disease
