# Supplementary material for: Association of the dietary copper intake with all-cause and cardiovascular mortality: A prospective cohort study
Source: PLoS One. 2023 Oct 13;18(10):e0292759. doi: 10.1371/journal.pone.0292759 (PMC10575518; doi:10.1371/journal.pone.0292759)
Supplement: S2 Table — (DOC) [file pone.0292759.s002.doc]

|  | Level | HR (95%CI) | P |
| --- | --- | --- | --- |
| Age |  | 1.11 (1.11,1.12) | <0.001 |
| Sex | Female | 1 |  |
|  | Male | 1.28 (1.13,1.44) | <0.001 |
| Race/ethnicity | Mexican American | 1 |  |
|  | Non-Hispanic Black | 2.15 (1.47,3.14) | <0.001 |
|  | Non-Hispanic White | 2.42 (1.68,3.47) | <0.001 |
|  | Other Race | 1.19 (0.77,1.82) | 0.043 |
| Education | College or above | 1 |  |
|  | High school or equivalent | 1.93 (1.66,2.24) | <0.001 |
|  | Less than high school | 3.66 (2.97,4.52) | <0.001 |
| Marital status | Married | 1 |  |
|  | Never married | 0.49 (0.39,0.61) | <0.001 |
|  | Separated | 2.89 (2.54,3.29) | <0.001 |
| Family income-poverty ratio | <1.0 | 1 |  |
|  | 1.0-3.0 | 1.28 (1.06,1.56) | 0.013 |
|  | >3.0 | 0.54 (0.44,0.66) | <0.001 |
| BMI |  | 1.02 (1.01,1.04) | 0.003 |
| copper |  | 0.70 (0.60,0.83) | <0.001 |
|  | Q1 (<0.8) | 1 |  |
|  | Q2 (≥0.8 to <1.1) | 0.80 (0.69,0.94) | 0.006 |
|  | Q3 (≥1.1 to <1.5) | 0.70 (0.59,0.83) | <0.001 |
|  | Q4 (≥1.5) | 0.44 (0.35,0.56) | <0.001 |
| Smoking status | Never | 1 |  |
|  | Former | 2.01 (1.73,2.32) | <0.001 |
|  | Now | 1.07 (0.89,1.29) | 0.480 |
| Alcohol use | Never | 1 |  |
|  | Mild | 0.76 (0.61,0.93) | 0.009 |
|  | Moderate | 0.32 (0.24,0.43) | <0.001 |
|  | Heavy | 0.25 (0.17,0.37) | <0.001 |
|  | Former | 1.48 (1.20,1.83) | <0.001 |
| DM | No | 1 |  |
|  | DM | 4.70 (4.11,5.37) | <0.001 |
|  | IFG | 2.43 (1.88,3.15) | <0.001 |
|  | IGT | 2.11 (1.58,2.83) | <0.001 |
| Hypertension |  | 5.77 (4.96,6.70) | <0.001 |
| Hyperlipidemia |  | 1.98 (1.70,2.31) | <0.001 |
| CVD |  | 1.03 (0.91,1.17) | <0.001 |
| CKD |  | 8.49 (6.96,10.40) | <0.001 |

S2 Table. Weighted univariate cox regression model for cardiovascular mortality.

Abbreviations:

HR, hazard ratio

CI, confidence interval

BMI, the body-mass index is determined as follows: the weight in kilograms (Kgs) / (height in square meters (m^2^)

DM, diabetes mellitus

IFG, impaired fasting glycaemia

IGT impaired glucose tolerance

CVD, cardiovascular disease

CKD, chronic kidney disease
