# Supplementary material for: Association of the dietary copper intake with all-cause and cardiovascular mortality: A prospective cohort study
Source: PLoS One. 2023 Oct 13;18(10):e0292759. doi: 10.1371/journal.pone.0292759 (PMC10575518; doi:10.1371/journal.pone.0292759)
Supplement: S3 Table — (DOC) [file pone.0292759.s003.doc]

| Copper | Q1 (<0.8) | Q2 (≥0.8 to <1.1) | Q3 (≥1.1 to <1.5) | Q4 (≥1.5) | *P* Value for Trend | *P* Value for Interaction |
| --- | --- | --- | --- | --- | --- | --- |
| All- cause mortality |  |  |  |  |  |  |
| Age, years |  |  |  |  |  |  |
| ≥65 | 1 | 0.95 (0.86-1.05) 0.317 | 0.84 (0.75-0.94) 0.002 | 0.77 (0.66-0.90) <0.001 | <0.001 | 0.087 |
| <65 | 1 | 0.80 (0.68-0.95) 0.012 | 0.83 (0.67-1.01) 0.062 | 0.78 (0.61-0.99) 0.040 | 0.062 |  |
| Sex |  |  |  |  |  |  |
| Female | 1 | 0.90 (0.79-1.02) 0.095 | 0.83 (0.73-0.96) 0.011 | 0.83 (0.67-1.02) 0.074 | 0.022 | 0.058 |
| Male | 1 | 0.93 (0.80-1.08) 0.321 | 0.93(0.80-1.09) 0.363 | 0.91 (0.76-1.08) 0.257 | 0.299 |  |
| Diabetes |  |  |  |  |  |  |
| No | 1 | 0.90 (0.80-1.00) 0.053 | 0.92 (0.81-1.05) 0.202 | 0.81 (0.70-0.94) 0.006 | 0.006 | 0.053 |
| Yes | 1 | 0.98 (0.85-1.13) 0.744 | 0.83 (0.70-0.99) 0.042 | 0.97 (0.81-1.17) 0.776 | 0.375 |  |
| Hypertension |  |  |  |  |  |  |
| No | 1 | 0.81 (0.69-0.95) 0.009 | 0.81 (0.68-0.97) 0.018 | 0.77 (0.62-0.95) 0.015 | 0.010 | 0.460 |
| Yes | 1 | 0.95 (0.86-1.05) 0.337 | 0.89 (0.80-1.01) 0.062 | 0.92 (0.80-1.05) 0.223 | 0.139 |  |
| Cardiovascular mortality |  |  |  |  |  |  |
| Age, years |  |  |  |  |  |  |
| ≥65 | 1 | 0.92 (0.77-1.09) 0.315 | 0.85 (0.72-1.01) 0.072 | 0.72 (0.55-0.92) 0.010 | 0.005 | 0.052 |
| <65 | 1 | 0.85 (0.58-1.24) 0.392 | 0.99 (0.67-1.47) 0.964 | 0.71 (0.43-1.15) 0.163 | 0.230 |  |
| Sex |  |  |  |  |  |  |
| Female | 1 | 0.88 (0.69-1.12) 0.294 | 0.84 (0.67-1.07) 0.155 | 0.69 (0.49-0.96) 0.028 | 0.007 | 0.880 |
| Male | 1 | 1.00 (0.78-1.28) 0.980 | 1.11 (0.85-1.45) 0.449 | 0.92 (0.67-1.25) 0.579 | 0.693 |  |
| Diabetes |  |  |  |  |  |  |
| No | 1 | 0.89 (0.72-1.10) 0.271 | 1.02 (0.81-1.29) 0.837 | 0.76 (0.58-1.00) 0.053 | 0.134 | 0.345 |
| Yes | 1 | 0.95 (0.74-1.20) 0.653 | 0.84 (0.63-1.14) 0.264 | 0.76 (0.51-1.12) 0.169 | 0.127 |  |
| Hypertension |  |  |  |  |  |  |
| No | 1 | 0.77 (0.50-1.12) 0.256 | 0.91 (0.61-1.36) 0.642 | 0.61 (0.38-0.97) 0.039 | 0.050 | 0.480 |
| Yes | 1 | 0.96 (0.82-1.13) 0.622 | 0.95 (0.80-1.14) 0.603 | 0.88 (0.69-1.14) 0.333 | 0.313 |  |

S3 Table. Weighted multivariable-adjusted hazard ratios for the association between quartiles of copper and all-cause mortality and cardiovascular mortality. by subgroups.

Data are hazard ratio (95% CI)

Models are adjusted for age, sex, ethnicity, family income-poverty ratio level, education, marital status, smoking status, diabetes, hypertension, CVD, and CKD except the subgroup variable itself.

Abbreviations:

HR, hazard ratio

CI, confidence interval

CVD, cardiovascular disease

CKD, chronic kidney disease
